# Autophagy in Neuroinflammation: A Focus on Epigenetic Regulation

**DOI:** 10.14336/AD.2023.0718-1

**Published:** 2024-04-01

**Authors:** Yu Chen, Junren Chen, Ziwei Xing, Cheng Peng, Dan Li

**Affiliations:** State Key Laboratory of Southwestern Chinese Medicine Resources, School of Pharmacy, Chengdu University of Traditional Chinese Medicine, Chengdu, China.

**Keywords:** Neuroinflammation, microglia, autophagy, epigenetic, central nervous system diseases

## Abstract

Neuroinflammation, characterized by the secretion of abundant inflammatory mediators, pro-inflammatory polarization of microglia, and the recruitment of infiltrating myeloid cells to foci of inflammation, drives or exacerbates the pathological processes of central nervous system disorders, especially in neurodegenerative diseases. Autophagy plays an essential role in neuroinflammatory processes, and the underlaying physiological mechanisms are closely correlated with neuroinflammation-related signals. Inhibition of mTOR and activation of AMPK and FOXO1 enhance autophagy and thereby suppress NLRP3 inflammasome activity and apoptosis, leading to the relief of neuroinflammatory response. And autophagy mitigates neuroinflammation mainly manifested by promoting the polarization of microglia from a pro-inflammatory to an anti-inflammatory state, reducing the production of pro-inflammatory mediators, and up-regulating the levels of anti-inflammatory factors. Notably, epigenetic modifications are intimately associated with autophagy and the onset and progression of various brain diseases. Non-coding RNAs, including microRNAs, circular RNAs and long noncoding RNAs, and histone acetylation have been reported to adjust autophagy-related gene and protein expression to alleviate inflammation in neurological diseases. The present review primarily focuses on the role and mechanisms of autophagy in neuroinflammatory responses, as well as epigenetic modifications of autophagy in neuroinflammation to reveal potential therapeutic targets in central nervous system diseases.

## 1. Introduction

Neuroinflammation refers to an inflammatory immune response to a variety of endogenous or exogenous stimuli such as pathogens and misfolded proteins within the central nervous system (CNS), which contributes critically to the pathogenicity and progression of almost all neurological disorders, such as Alzheimer's disease (AD) and Parkinson's disease (PD) [1-4]. The principal characteristic of neuroinflammation is the overproduction of inflammatory factors in the CNS, including cytokines like TNF-α, IL-18 and IL-6, chemokines such as CCL2, CCL5 and CXCL3, and small-molecule messengers including NO and ROS [5-7]. Immune cells, particularly microglia, the primary resident innate immune cells in the CNS, are main sources of inflammatory mediators. And excessive pro-inflammatory mediators trigger excitotoxicity, apoptosis, and necrosis induing neuronal damage, and disrupt the permeability of blood-brain barrier that leading to neutrophil infiltration, thus causing or further aggravating CNS diseases [8, 9]. Moreover, hyperstimulation of microglia with a reduced ability to phagocytose and degrade misfolded proteins, promotes the aggregation of α-synuclein, β-amyloid (Aβ) and tau, to form Lewy bodies, amyloid plaques, and tau neurofibrillary tangles [10-12]. At present, neuro-inflammatory inhibitors have been widely explored as potential tools for treating neurological diseases [13], that mainly focus on these targets like TLRs, NF-κB, NLRP3 inflammasome, etc. [14-16]. Noteworthy, microglia could recruit Aβ to autophagic vacuoles for degradation and inhibit Aβ-induced activation of NLRP3 inflammasome, indicating that the ability of microglia in phagocytosis and degradation of abnormal proteins is closely associated with autophagy-related mechanisms in neuroinflammation [17, 18]. And recently, many studies have reported the participation of autophagy in neuroinflammation and support an autophagic regulation role in the pathogenesis of neuroinflammation-related CNS disorders [19, 20].

**Figure 1.**
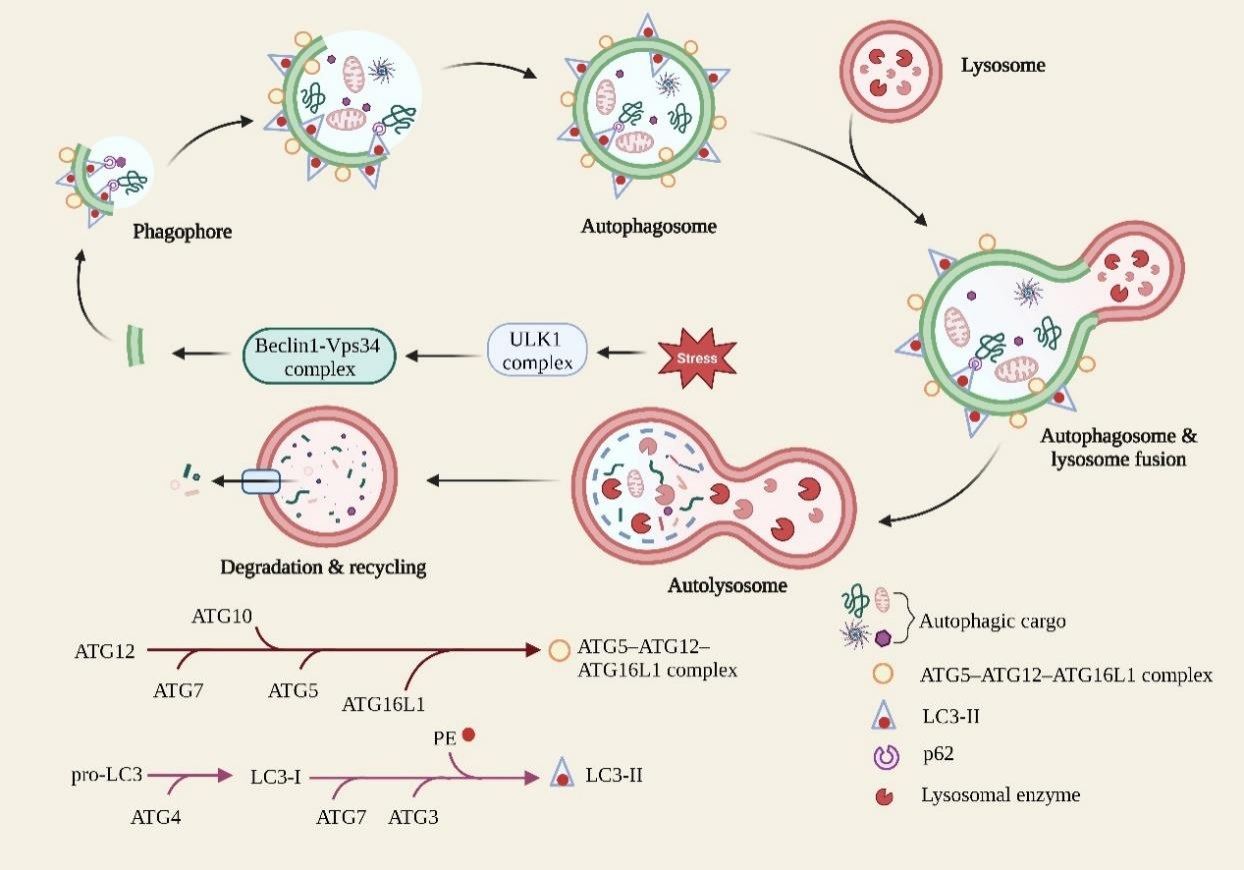
**Process of autophagy**. Under stress conditions, Beclin1-Vps34 complex stimulated by ULK1 complex induces the initiation of autophagy and promotes formation of phagophore. Phagophores surround autophagic cargoes, then expand to form autophagosomes and fuse with lysosomes to form autolysosomes, where sequestered cargoes are digested and degraded.

Autophagy is a strictly regulated process of cellular degradation to maintain cellular and intracellular homeostasis, and can be activated by pressure conditions like hypoxia, insufficient nutrition and infection [21]. Through autophagy, cytoplasmic components, such as intracellular pathogens, impaired organelles as well as misfolded proteins, are transported to the lysosome for degradation [22, 23]. Despite autophagy exhibiting cytoprotective properties preventing a variety of diseases, such as neurodegenerative diseases, in some cases autophagy also exerts harmful effects through cytocidal effects on normal cells [24]. Depending on the different ways in which cargoes are transported to the lysosome, autophagy is commonly categorized into macroautophagy, microautophagy and chaperone-mediated autophagy among eukaryotic cells [21]. In macroautophagy (hereafter termed autophagy) (Fig. 1), cytoplasmic cargo is captured by phagophore and forms a double-membrane autophagosome that subsequently fuses with the lysosome for degradation [25]. In the above process, autophagy is initiated from ULK1 complex-mediated phosphorylation of Beclin1-Vps34 complex, promoting formation of phagophore and autophagosome, whose membranes are formed by LC3-II binding to lipid phosphatidylethanolamine (PE) and autophagy-associated proteins (ATG) such as ATG5, ATG12 and ATG16L1 [23]. Autophagy plays a vital role in neurological disorders, like AD, PD, and multiple sclerosis, probably through mediating inflammatory signals [26]. *In vitro*, defective autophagy of microglia exacerbates the production of pro-inflammatory mediators induced by lipopolysaccharide (LPS) or Aβ and leads to apoptosis of neurons in the co-culture state. And *in vivo*, impaired microglia autophagy similarly exacerbates neuron loss and neuroinflammatory response caused by 1-methyl-4-phenyl-1,2,3,6-tetrahydropyridine (MPTP) or Aβ fibrils [18, 27]. Moreover, autophagy deficiency exacerbates CNS diseases due to NLRP3 inflammasome activation, inflammatory cytokines release and accumulation of abnormal proteins [18, 27, 28]. Thus, the role of autophagy as a core mediator of neuroinflammation in CNS diseases deserves more attention.

Epigenetic modification of autophagy is a new critical regulator to maintain cellular homeostasis via modifying autophagy-related gene and protein expression, thereby influencing subsequent autophagic flux, while epigenetic dysregulation leads to autophagic dysfunction, that induces a variety of CNS diseases [24, 29]. In addition, various epigenetic changes, including DNA methylation, histone modifications and non-coding RNAs, are involved in CNS diseases, such as PD, stroke and neuropathic pain via influencing multiple inflammatory signals to modulate microglia polarization and the production of inflammatory mediators [30-33]. However, epigenetic mechanisms of autophagy that regulate neuroinflammation and related disorders have not been systematically described so far. Therefore, in this review, the role and mechanisms of autophagy in neuroinflammatory responses, as well as epigenetic modifications of autophagy in neuroinflammation-related neurological disorders were systemically reviewed to unveil potential therapeutic targets.

## 2. Autophagy in neuroinflammation and related CNS diseases

### 2.1 mTOR and autophagy

mTOR is a classic kinase that modulates cell growth and proliferation with a critical effect in the autophagy regulation of microglia phenotype. In LPS-stimulated N9 microglia, pro-inflammatory mediators like TNF-α, IL-6, and iNOS levels were up-regulated, and autophagy were suppressed by enhancing p62 expression and reducing LC3-II/I ratio and ATG5, Beclin1 and Vps34 expression with activation of PI3k/AKT/mTOR signaling pathway. However, mTOR inhibitor rapamycin alleviated LPS-induced neuroinflammation and promoted autophagy in N9 microglia and hippocampus and cortex from neuroinflammatory mice [34]. Additionally, ablation of neural sphingosine-1-phosphate (S1P) lyase 1 (SGPL1) markedly activated primary microglia, increased pro-inflammatory cytokines levels and inhibited autophagy via mTOR signal. And LPS further enhanced IL-6 release and autophagy inhibition, which was reversed by rapamycin and SIPR2 inhibitor in primary microglia from SGPL1 ablated mice [35]. Another study reported that in hypoxia-ischemia and LPS-induced cerebral palsy mouse pups, rapamycin suppressed microglia activation, decreased HIF-1α and cleaved Caspase-3 expression, up-regulated Beclin1 expression and LC3-II/I ratio and suppressed phosphorylation of p70S6K and S6 in the brain tissue [36]. Interestingly, inhibition of mTOR signaling not only inhibited microglia activation, but also promoted polarization of microglia toward an anti-inflammatory state (M2) by activating autophagy. TNF-α, an important inflammatory cytokine, significantly reduced the expression of M2 markers, increased the expression of M1 markers, promoted the phosphorylation of AKT and mTOR, and impaired autophagic flux in microglia, which were reversed by AKT inhibitor perifosine. Moreover, autophagy activation by rapamycin enhanced M2 markers and reduced M1 markers’ gene expression, whereas autophagy inhibition with 3-methyladenine (3-MA) or ATG5 siRNA aggravated TNF-α-stimulated M1 polarization in microglia [37]. Besides, in primary microglia from SOD1-G93A mice, P2X7 activation using 2’-3’-O-(benzoyl-benzoyl) ATP increased M2 markers expression and activated autophagy via inhibiting mTOR phosphorylation [38]. Another study reported that Sestrin2 promoted microglial M2 polarization to provide neuroprotection after oxygen and glucose deprivation (OGD) and re-oxygenation (OGD/R) in BV2 cells, characterized by restoration of autophagic flux with up-regulation of LAMP2 and inhibition of mTOR phosphorylation, which were further confirmed in the middle cerebral artery occlusion (MCAO)-induced mice [39]. In sum, inhibition of mTOR signaling might suppress the activation of microglia and promote microglia polarization toward M2 state by activating autophagy.

Multiple antagonists or inhibitors also promote autophagy through mTOR signal to alleviating neuroinflammation in CNS diseases. Metabotropic glutamate receptor 5 (mGluR5) antagonist MPEP alleviated neuroinflammatory response, promoted autophagy with increased expression of LC3-II and Beclin1, and decreased expression of p62 via PI3K/AKT/mTOR signal in AD mice [40]. BAY61-3606, a spleen tyrosine kinase (SYK) inhibitor, increased autophagic flux as well as inhibited mTOR phosphorylation, resulting in reduced neuronal and synaptic loss and pro-inflammatory cytokines secretion in Tau P301S mice [41]. Moreover, MSDC-0160, a mitochondrial pyruvate carrier (MPC) inhibitor, improved motor dysfunction, protected nigrostriatal neurons from inflammation in MPTP-stimulated PD mice and engrailed1 genetic PD mice, evidenced by inhibiting mTOR signal and restoring autophagy [42]. Altogether, blockade of mTOR signaling pathway observably attenuates neuroinflammation and related brain diseases via promoting autophagy.

### 2.2 AMPK and autophagy

AMPK is demonstrated to directly promote autophagy by phosphorylating autophagy-associated proteins, such as mTOR, ULK1 and Beclin1-Vps34 complexes. LKB1-AMPK pathway was suppressed in LPS-induced primary microglia, accompanied by the activation of M1 microglia, up-regulation of pro-inflammatory cytokines levels, and inhibition of autophagy with decreased p62 expression and increased LC3-II/I ratio, as well as Beclin1 and ATG5 expression. Whereas knockdown of PPARγ and its antagonist activated LKB-AMPK signal to inhibit LPS-induced changes in primary microglia [43]. Additionally, AMPK-mTOR-p70S6K axis participated in the anti-neuroinflammatory effects of PNU282987, an alpha7 nicotinic acetylcholine receptor (α7nAChR) activator. PNU282987 down-regulated the levels of pro-inflammatory cytokines through increasing autophagy flux in LPS-induced BV2 cells and in spinal cord and spleen tissue from experimental autoimmune encephalomyelitis (EAE) mice. Nevertheless, an AMPK inhibitor compound C as well as blockade of autophagy by ATG5 siRNA, bafilomycin A1 and 3-MA attenuated the effects of PNU282987 [44]. Moreover, inhibition of AMPK pathway also activated microglia and astrocytes, increased the production of TNF-α, NLRP3 and ASC, and reduced autophagy flux in the hippocampus from mice with high-fat feeding-triggered depression, while autophagy activator attenuated the above changes caused by high fat feeding [45]. Thus, these results suggest that activation of AMPK signal could enhance autophagy, thereby inhibiting microglia M1 polarization and NLRP3 inflammasome activation, and subsequently reducing the expression of pro-inflammatory mediators in neuroinflammation-related CNS disorders.

### 2.3 HMGB1 and autophagy

HMGB1, as an autophagy sensor, is a DNA-binding protein in the nucleus or translocated into the cytoplasm during cellular stress, which were secreted from necrotic neurons to activate microglia and other immune cells to drive neuroinflammation in CNS diseases [46]. Knockdown of HMGB1 exacerbated the decrease of cell viability, increased apoptosis and cleaved Caspase-3 production, and reduced autophagic flux with up-regulation of p62 and down-regulation of LC3-II/I ratio via perturbing Beclin1-Bcl-2 complex formation in emulsified isoflurane-stimulated SH-SY5Y and PC12 cells [47]. Additionally, HMGB1 siRNA decreased pro-inflammatory cytokines secretion and the number of LC3-positive cells, and reduced the expression of TLR4, MyD88, Beclin1, and ATG5 in ipsilateral striatum of rats after intracerebral hemorrhage (ICH) [48]. Moreover, exogenous disulfide HMGB1 impaired mitophagy flux and activated NF-κB signaling pathway, which were reversed by rapamycin in microglia. *In vivo*, HMGB1 siRNA attenuated microglia M1 activation and reduced the transcription and translation of RAGE, a HMGB1 receptor, in the rostral ventrolateral medulla of stress-induced hypertension in mice. And microglia-specific deletion RAGE suppressed the activation of M1 microglia, reduced autophagosomes accumulation and the expression of mitochondrial reactive oxygen species (mtROS), and impaired lysosomal function in stressed mice [49]. To sum up, HMGB1 might have a dual regulatory role with regulating autophagy to neural injury in CNS diseases.

### 2.4 NLRP3 inflammasome and autophagy

NLRP3 inflammasome has been illustrated to be activated in neuroinflammation and related CNS diseases, especially in PD. NLRP3 gene knockout decreased pro-inflammatory cytokines secretion and Caspase1 expression, improved nigral autophagy, that manifested by an increase in LC3-II expression and a decrease in p62 level, and suppressed nigral dopaminergic degeneration, striatal dopamine (DA) deletion, glial activation and nigral α-synuclein aggregation in chronic MPTP-treated PD mice [50]. ATG5 and ATG7 are among the core mediators of autophagosome biogenesis, and their absence induced NLRP3 inflammasome activation. Deletion of ATG5 worsened LPS-induced neuro-inflammation, including enhancing the level of IL-1β, iNOS and TNF-α, and reducing the expression of Arg-1 and Ym-1 in BV2 cells, and also promoted the activation of NLRP3 inflammasome in LPS plus ATP-stimulated microglia. And microglia-specific knockdown of ATG5 aggravated motor dysfunction and dopaminergic neurodegeneration, enhanced microgliosis and astrogliosis, exacerbated NLRP3 inflammasome activation in the substantia nigra (SN) of MPTP-induced PD mice [27]. Moreover, ATG5 knockout in microglia caused PD-like motor and cognitive dysfunction, and induced neuronal damage, increased the production of pro-inflammatory mediators, as well as promoted NLRP3 inflammasome activation and PDE10A expression in the striatum of mice, while inhibition of NLRP3 using MCC950 rescued microglia activation and neuronal loss [51]. Apart from ATG5, ATG7 knockout also promoted the secretion of IL-1β, IL-6 and TNF-α, and cleaved Caspase-1 and ASC expression in BV2 cells. And in ATG7 conditional knockout mice, loss of ATG7 function triggered microgliosis and increased pro-inflammatory cytokines genes levels in sorted microglia from the brain of mice [52]. Taken together, the results of *in vitro* and *in vivo* models of PD indicate that autophagy interacts with NLRP3 inflammasome, and the enhancement of autophagy in microglia might inactivate NLRP3 inflammasome, thus inhibiting the production of pro-inflammatory mediators to alleviate the symptoms of PD.

Autophagy attenuates neuroinflammatory response by inhibiting the activation of NLPR3 inflammasome in subarachnoid hemorrhage (SAH), migraine and other CNS disorders. LPS plus ATP stimulation increased NLRP3 inflammasomes activation, and inhibited autophagy with a reduction of Beclin1 and LC3-II proteins expression in primary microglia [53]. NLRP3 inflammasomes were also activated in SAH, with increased levels of IL-1β and IL-18, and neurological dysfunction via suppressing autophagy, which were partly reversed by growth arrest-specific 6, a MerTK activator [54]. Besides, autophagy inducer, rapamycin and P2X7R antagonist, Brilliant Blue G, suppressed microglia and NLRP3 inflammasome activation, decreased levels of pro-inflammatory cytokines and promoted autophagy in the trigeminal nucleus caudalis from nitroglycerin-induced chronic migraine mice [55]. Additionally, NLRP3 inhibitor, MCC950 and mitophagy inducer, rapamycin, mitigated neurological damage, decreased pro-inflammatory factors levels, reduced the expression of NLRP3, ASC and Caspase-1, promoted the expression of PINK1 and LC3-II, and attenuated mitochondrial dysfunction with increased mitochondrial DNA, mitochondrial membrane potential and ATP level, as well as decreased ROS production in OGD-induced primary cortical neuron and cortical tissue of traumatic brain injury (TBI) mice [56]. Thus, enhanced autophagy of microglia and mitophagy of neurons could notably inhibit NLRP3 inflammasome activation thereby downregulating pro-inflammatory mediators’ levels in migraine, TBI and other CNS disorders.

Additionally, autophagic inhibitor 3-MA treatment worsened ethanol-induced memory impairment, while autophagy activation with rapamycin suppressed IL-1β and IL-18 genes transcription and NLPR3, pro-Caspase-1 and Caspase-1 proteins expression in ethanol-induced microglia and mice [19]. Moreover, NLRP3 inflammasomes were activated by anesthesia and surgery, along with cognitive impairment, microglia activation, increased pro-inflammatory cytokines expression, and impaired mitophagic flux, which were inverse by triggering receptor expressed on myeloid cells 2 (TREM2) in mice [57]. Besides, LPS interfered mitochondrial function and mitophagy *via* activating NLRP3 inflammasomes in N9 microglia and hippocampus of *db/db* mice, but exendin-4, a GLP-1R activator, alleviated above changes and relieved depression-like behaviors in diabetic mice [58]. Furthermore, autophagy agonist rapamycin improved cognitive dysfunction, inhibited the activation of prefrontal cortex microglia, decreased generation of pro-inflammatory cytokines and inactivated NLRP3 inflammasome via enhancing autophagy in sevoflurane exposure-induced rats [59]. In general, autophagy might inhibit the activation of NLRP3 inflammasome and reduce the production of pro-inflammatory mediators to alleviate neuroinflammation and thus attenuate memory and cognitive behavioral deficits.

### 2.5 TLRs and autophagy

TLRs, abundantly expressed in CNS diseases, mainly activate neuroinflammatory response by targeting autophagy signals [60, 61]. TLR2 triggered by peptidoglycan significantly induced over-activation of autophagy with increased ratio of LC3-II/I and Beclin1 expression in BV2 cells. While both the TLR2 gene knockout and its antagonist CU-CPT22 dramatically inhibited autophagy levels and promoted microglia M2 polarization, including augmentation of CD206, Arg-1 and IL-10 in peptidoglycan-induced BV2. Further, TLR2 gene knockout also inhibited autophagy and elevated microglia survival in the hippocampus from peptidoglycan-induced mice [62]. Besides, erythrocyte lysis and LPS increased the secretion of pro-inflammatory factors and TLR4 expression, and induced microglial autophagy in primary microglia, which were inhibited by knockdown of TLR4 gene [63]. Moreover, TLR4 knockdown improved TBI-induced brain damage and neuroinflammation via inhibiting excessive autophagy in the hippocampus, which were abolished by autophagy inducer rapamycin [64]. Other study also found that TLR4 antagonist improved behavioral impairment, brain edema and neurons loss, and suppressed MyD88/NF-κB signal and autophagy in the hippocampus of TBI rats [65]. Altogether, these investigations indicate that the inhibition of TLRs, including TLR2 and TLR4, might suppress the over-activation of autophagy to alleviate neuroinflammation in CNS diseases.

### 2.6 FOXO1 and autophagy

Recently, some evidences suggested that FOXO1 may regulate autophagy to improve neuroinflammation. In the brain cortex of mice, LPS exposure inhibited FOXO1 signaling, promoted microglia M1 state activation, and suppressed autophagy with decreased of LC3, Beclin-1, ATG5, ATG7 and ATG12 transcription and translation. LPS also result in imbalance of brain renin-angiotensin system, including up-regulation of the expression of angiotensin II, angiotensin-converting enzyme (ACE), angiotensin type (AT) 1, AT2, and MasR proteins, as well as down-regulation of ACE2 expression. AVE, the selective MasR agonist, reversed LPS-induced above changes and exhibited neuroprotective actions, while FOXO1 inhibitor or autophagy inhibitor compromised the anti-neuroinflammatory actions of AVE in mice [66]. Collectively, this indicates that the interaction between FOXO1 and autophagy promotes M1 to M2 polarization of microglia to alleviate neuroinflammatory response.

### 2.8 Others

*In vitro* and *in vivo* models of cerebral ischemia, in addition to mTOR and NLRP3 inflammasome, PRNP, sphingosine kinase 1 (SphK1), DJ-1 and other signals participate in the regulation of neuroinflammation through autophagy as well. In primary microglia from wild-type mice, autophagy inhibition with 3-MA and bafilomycin A1 further aggravated OGD/R-induced inflammatory response, induced microglial M1 polarization and increased the secretion of pro-inflammatory cytokines. And PRNP, the gene encoding the prion protein, its knockout suppressed microglia M2 polarization, further aggravated M1 polarization and shortened the accumulation of LC3-II, while microglia overexpressing PRNP showed increased level of LAMP1 [67], which suggested that PRNP attenuated OGD/R-induced inflammation by augmenting and extending autophagy activation. Besides, OGD/R induced the activation of autophagy and the increase of SphK1 in primary microglia, while SphK1 knockdown significantly inhibited OGD/R-induced autophagy and thereby protected against neuronal damage via suppressing TRAF2 expression. Further, SphK1 knockdown reduced apoptotic neuronal death and microglial autophagy in peri-infarct cortical tissue [68]. Moreover, DJ-1 siRNA decreased the expression of Sirt1 and suppressed ATG5-ATG12-ATG16L1 complex expression and conjugation to promote microglia polarization from M2 to M1 state during MCAO/reperfusion-stimulated I/R injury, and the inhibition of Sirt1 exacerbated the inhibitory effects of DJ-1 interference [69].

Autophagy is impaired in AD, PD and TBI, which could be repaired by the inhibition of 12/15Lipoxygenase (12/15LO), LRRK2, and STING. Over-expressed 12/15LO worsened behavioral deficits, increased Aβ peptides levels, and reduced LC3-II/I ratio and the expression of ATG7 and ATG12-ATG5, resulting in serious neuroinflammation in 3xTg mice [70]. Besides, LPS induced dopaminergic neuron loss, a-synuclein accumulation and autophagic impairment with more lysosomes and autolysosomes, a continuous increase in p62 and an increase followed by a decrease in LC3-II and HDAC6 in the midbrain of PD mice [71]. Additionally, following manganese exposure or LPS stimulation in mice, LRRK2 siRNA inhibited the loss of dopaminergic neurons, alleviated neuroinflammation and autophagy dysfunction [72]. What's more, the expression of STING elevated in post-mortem human TBI brain and TBI mice brain. And genetic ablation of STING reduced lesion volumes, glia activation and pro-inflammatory mediator’s gene expression, inhibited type-I IFN signaling including down-regulation of IFN-α, IFN-β and IRF3 expression and promoted autophagic flux in the brain from TBI mice [73]. Additionally, pifithrin-α, a p53 inactivator, improved neurological functional outcomes, inhibited astrocytes and microglia activation, down-regulated the levels of pro-inflammatory cytokines via enhancing autophagy in striatal tissues of TBI rats [74].

Intriguingly, autophagy is over-activated in some CNS disorders, so its inhibition might mitigate neuroinflammation instead. During S. pneumonia infection, BV2 cells showed increased LDH, IL-6, and IL-18 levels, and up-regulation of NOD2 expression. And ablation of NOD2 partly reversed above changes to improve neuroinflammation via suppressing TAK1/NF-κB signal and hyperactivated autophagy [75]. Besides, autophagy was induced accompanied by an accumulation of LC3 dots and autophagosomes, and an increase expression of LC3-II protein, which were reversed by siRNA targeting ATG5, Beclin1, ATG9 and ATG12 in streptococcus suis serotype 2-infected BV2 cells and mice [76]. Moreover, ULK1 gene knockout alleviated TBI-induced behavior impairment, reduced neuro-inflammation and apoptosis along with inhibition of microglia and astrocytes activation, an increase in Bcl-2 expression, a decrease in proinflammatory cytokines and the expression of Bax, cytochrome c, cleaved Caspase-3 and cleaved PARP in hippocampus. ULK1 gene knockout also inhibited the overactivation of autophagy via inhibiting the phosphorylation of p38 and JNK in hippocampus from TBI-stimulated mice, which were further confirmed in LPS-treated primary astrocyte with or without ULK1 siRNA transfection [77]. Inhibition of autophagy with bafilomycin A1 ameliorated chronic unpredictable mild stress-induced depressive-like behaviors, inhibits apoptosis, reduced pro-inflammatory cytokines levels, increased synaptic plasticity-associated proteins SYP and PSD95 expression in brains from depression rats [78].

In summary, apart from mTOR, AMPK and NLRP3, other signals, such as Sphk1, DJ-1, LRRK2 could modulate autophagy to participate in microglia polarization as well as inflammatory mediators’ production, thus affecting neuroinflammatory processes in cerebral ischemia, AD, TBI and other CNS diseases.

## 3. Epigenetic regulation of autophagy in neuroinflammation and related CNS diseases

### 3.1 Non-Coding RNAs Modification

Non-coding RNAs, such as microRNAs (miRNAs), circularRNAs (circRNAs) and long non-coding RNAs (lncRNAs), represent key regulators of autophagy and are able to mediate the occurrence and progression of brain disorders by modulating autophagy [79]. The role of non-coding RNAs modifications in modulating autophagy in neuroinflammation-associated CNS diseases are well shown in Table 1.

**Table 1:** Epigenetic regulation of autophagy in neuroinflammation and related CNS diseases.

Epigenetic regulation	Molecular biological tool	Dosage	Model	Index	Disease	Ref
microRNAs	miR-124 inhibitor	100 nM	LPS-induced BV2 cells	↑: TNF-α, IL-6	PD	[80]
	miR-124 mimic	50 nM		↓: TNF-α, IL-1β, p62, p-p38 ↑: LC3-II, LC3-II/I		
	p62 siRNA	100 nM		↓: TNF-α, IL-1β, iNOS, p62, p-p38		
	p38 inhibitor	1, 10, 50 µM		↓: TNF-α, IL-1β, iNOS ↑: LC3-II, LC3-II/I		
	miR-124 agomir	20 nM/5µL, i.c.v.	MPTP-induced mice	↓: IL-1β, Iba-1, p62, p-p38 ↑: LC3-II, LC3-II/I		
	miR-3473b inhibitor		LPS-induced BV2 cells	↓: TNF-α, IL-1β	PD	[81]
	TREM2 plasmid			↓: TNF-α, IL-1β ↑: p-ULK1		
	ULK1 plasmid			↑: LC3-II, LC3-II/I		
	miR-3473b antagomir	20 nM/5 µL, i.c.v.	MPTP-induced mice	↓: Iba-1 ↑: LC3-II, LC3-II/I, p-ULK1, TREM2		
	double transgenic APP/PS1		Aβ (1-42)-induced primary microglia isolated from 5xFAD mice	↓: Aβ degradation	AD	[82]
			primary microglia isolated from 5xFAD mice	↑: Iba-1, miR-17 ↓: ATG7, NBR1		
	ATG5 knockout		Aβ (1-42)-induced primary microglia isolated from ATG5^-/-^ mice	↓: Aβ degradation		
	miR-17 inhibitor	50 nM	Aβ (1-42)-induced primary microglia isolated from 5xFAD mice	↑: Aβ degradation, LC3 volume, NBR1		
	NBR1 siRNA	50 nM	Aβ (1-42)-induced human microglial cell line (HMC3)	↓: Aβ degradation		
	double transgenic APP/PS1		5xFAD mice	↑: Iba-1 ↓: NBR1		
	miR-17 antagomir	1.2 nM/4 µL, intracisternal magna injection		↑: NBR1 in microglia, ATG7 in microglia		
	miR-223 inhibitor		LPS-induced BV2 cells	↑: autophagosomes, LC3-II ↓: TIMM23	multiple sclerosis	[83]
	miR-223 mimic			↓: LC3 puncta, LC3-II ↑: TIMM23		
	miR-223 knockout		EAE mice	↓: clinical scores, infiltration of mononuclear cells, active microglia ↑: resting microglia, LC3 puncta		
	autophagy inhibitor: 3-MA	10 mg/kg, i.p.	EAE miR-223^-/-^ mice	↑: clinical scores		
	IL-6 siRNA	50 nM	OGD-activated microglia medium-induced PC12 cells	↓: apoptotic neurons, autophagosomes, LC3-II	ischemic brain injury	[84]
	autophagy inhibitor: Chloroquine	10 µM	recombinant rat IL-6-induced PC12 cells	↓: apoptotic neurons		
	miR-30d mimic	50 nM	OGD-activated microglia medium-induced PC12 cells	↓: apoptotic neurons, autophagosomes, cleaved Caspase-3, cleaved Caspase-9, LC3-II		
	autophagy inhibitor: Bafilomycin A1	10 nM	hemoglobin-induced primary hippocampal microglia	↓: TNF-α, IL-1β, IL-6	ICH	[85]
	autophagy activator: rapamycin	100 nM		↑: TNF-α, IL-1β, IL-6		
	pcDNA-mTOR	50 nM		↓: TNF-α, IL-1β, IL-6, LC3 punctate, autophagosomes, LC3-II		
	miR-144 inhibitor	50 nM		↓: TNF-α, IL-1β, IL-6 ↑: mTOR		
	miR-144 inhibitor	2 µg/2 µL, i.c.v.	ICH model mice	↓: cerebral water content, neurological deficit scores, TNF-α, IL-1β, IL-6, LC3-II/I, miR-144 ↑: mTOR	ICH	[86]
	pcDNA-mTOR	2 µg/2 µL, i.c.v.		↓: TNF-α, IL-1β, IL-6		
	miR-23b mimic		BV2 cells	↓: autophagosomes, autolysosomes, LC3-II, IPMK ↑: p62	ICH	[87]
			hemin-stimulated BV2 cells	↓: IL-1β, TNF-α, NO, iNOS, CCL2, IPMK, ↑: p-Akt, p-mTOR, miR-23b		
	miR-23b inhibitor		BV2 cells	↑: TNF-α, IL-1β, IL-6, NO, iNOS, CCL2, LC3-II ↓: p62		
			hemin-stimulated BV2 cells	↑: IPMK ↓: p-Akt, p-mTOR		
	lentivirus-miR-23b	5 μL 10^9^ transfection unit/mL, i.c.v.	ICH model rats	↓: brain water content, Iba-1^+^ cells, apoptotic cells, iNOS, CCL2, cleaved Caspase-3, Bcl-2 ↑: NeuN^+^ cells, miR-23b		
	lentivirus-miR-27a	1μL, i.c.v.	TBI model rats	↓: mNSS scores, brain water content, lession volume, histopathological changes, LC3-II, Beclin-1, FOXO3a ↑: p62	TBI	[88]
	FOXO3a siRNA	5 μL, (10^7^-10^8^ pfu/mL), i.c.v.		↓: mNSS scores, brain water content, lession volume, FOXO3a, Beclin-1 ↑: p62		
circularRNAs	miR124-2HG		methamphetamine-induced astrocytes	↓: GFAP, LC3-II, DDIT3, Sigmar1	neuroinflammation	[89]
	Sigmar1 siRNA		anti-miR124-2HG-induced astrocytes	↓: GFAP, LC3-II, DDIT3		
	anti-miR124-2HG lentivirus	2 μL 10^9^ viral genomes/μL, i.c.v.	mice	↑: GFAP, Sigmar1		
			Sigmar1 knockout mice	↓: GFAP		
	circHIPK2 siRNA		methamphetamine-induced astrocytes	↓: GFAP, LC3-II, DDIT3, Sigmar1		
			anti-miR124-2HG-treated astrocytes	↓: GFAP, LC3-II, DDIT3, Sigmar1		
		2 μL 10^9^ viral genomes/μL, i.c.v.	methamphetamine/LPS-induced mice	↓: GFAP, Sigmar1		
	circRS-7 siRNA	10 μL, intrathecal injection	CCI-induced neuropathic pain rats	↑: p62 ↓: IL-6, IL-12, TNF-α, Iba-1, Beclin1, LC3-I, LC3-II, circRS-7	Neuropathic pain	[90]
	miR-135a-5p inhibitor	10 μL, intrathecal injection		↑: Beclin1, LC3-I, LC3-II, miR-135a-5p ↓: p62		
long non-coding RNAs	lincRNA-Cox2 knockdown		LPS and ATP-induced microglia	↓: IL-1β, pro Caspase-1, ASC, lincRNA-Cox2 ↑: cleaved TRIF, LC3 puncta, LC3-II/I	neuroinflammation	[91]
			LPS-induced BV2 cells	↓: NLRP3, p65 recruitment to the NLRP3 and ASC gene promoter region, nuclear p65		
	NLRP3 siRNA	25 nM	LPS and ATP-induced lincRNA-Cox2 knockdown microglia	↑: LC3-II/I ↓: IL-1β		
	Caspase-1 siRNA	25 nM				
	TRIF siRNA	25 nM		↓: LC3 puncta, LC3-II/I		
	lincRNA-Cox2 shRNA	200 μL (titer ≥ 1x10^8^ IU/mL), i.v.	EAE model mice	↓: clinical score, infiltration of inflammatory cells, Iba-1, IL-1β ↑: LC3		
	lncRNA-HOTAIR siRNA		MPP^+^-stimulated SK-N-SH cells	↑: cell viability, apoptosis rate, LDH, ROS, IL-6, IL-1β, TNF-α, Bax, ATG10, lncRNA-HOTAIR, ↑: SOD, Bcl-2, miR-874-5p	PD	[92]
	miR-874-5p inhibitor		MPP^+^-stimulated lncRNA-HOTAIR knockdown SK-N-SH cells	↓: cell viability, SOD, Bcl-2 ↑: apoptosis rate, IL-6, IL-1β, TNF-α, LDH, ROS, ATG10, Bax		
	ATG10 overexpression vector		MPP^+^-stimulated miR-874-5p-treated SK-N-SH cells	↑: apoptosis rate, IL-6, IL-1β, TNF-α, LDH, ROS, Bax ↓: SOD, Bcl-2		
Histone Modification	Histone deacetylases inhibitor (uberoylanilide hydroxamic acid)	1 µM	sevoflurane-exposed primary hippocampal neurons	↑: H3 and H4 histone acetylation, LC3-II/I ↓: p62	Age-related cognitive impairment, neuroinflammation	[93]
	Histone deacetylases inhibitor (uberoylanilide hydroxamic acid)	50 mg/kg, i.p.	evoflurane-stimulated aged mice	↓: cognitive impairment, NLRP3, cleaved Caspase-1, IL-1β, p62 ↑: H3 and H4 histone acetylation, autophagosomes, LC3-II/I		
	Histone acetyltransferases inhibitor: anacardic acid	20 μM	CGRP-stimulated rat C6 astrocyte cells	↓: CX3CR1, IL-1β, H3K9ac, LC3-II	Neuropathic pain	[94]
	Histone acetyltransferases inhibitor: anacardic acid	20 μM, intrathecal injection	CCI-induced rats	↓: pain hypersensitivity, GFAP, CX3CR1, IL-1β, H3K9ac		
	CGRP antagonist: CGRP8- 37	2 μM, intrathecal injection				

“↑” indicates promotion, “↓” indicates inhibition.

#### 3.1.1 miRNAs

A study reported that *miR-124* inhibition aggravated LPS-induced TNF-α and IL-1β increase, and *miR-124* overexpression attenuated LPS-induced neuro-inflammation by directly targeting p62 and p38, and up-regulating the ratio of LC3-II/I in BV2 cells. Further, exogenous delivery of *miR-124* also alleviated neuroinflammatory response via activating autophagy in the substantia nigra par compacta (SNpc) of MPTP-induced PD mice [80]. Besides, inhibition of *miR-3473b* suppressed pro-inflammatory cytokines levels via directly targeting TREM2 and increasing the phosphorylation of ULK1 to promote autophagy in LPS-stimulated BV2 cells. And in the SNpc from MPTP-treated PD mice, MPTP up-regulated the expression of *miR-3473b*, inhibited autophagy, and exogenous delivery of *miR-3473b* antagomir reversed above changes [81]. Moreover, Aβ degradation was decreased in primary microglia from 5xFAD mice, autophagy-inhibited microglia and NBR1 knockdown human microglia line HMC3. And the expression of NBR1 and ATG7 was suppressed in 5xFAD microglia and in microglia of human AD brain slices, mainly in regions of high Aβ burden, whereas *miR-17* expression was increased in human AD brain slices. Further studies showed that *miR-17* inhibition promoted autophagy and Aβ degradation and up-regulated the expression of NBR1 in primary microglia from 5xFAD mice [82]. Another research reported that *miR-223* deficiency further increased the number of autophagic vacuoles and GFP-LC3 accumulation, promoted LC3 lipidation and decreased TIMM23 expression in LPS-induced BV2 cells, which were blocked by *miR-223* mimics via directly targeting ATG16L1 3′-UTR. And in EAE mice, *miR-223* knockout alleviated EAE symptoms and CNS neuroinflammation, inhibited microglia activation and augmented autophagy in brain microglia [83]. Interestingly, in some CNS disorders, autophagy over-activation leads to increased inflammation and microRNAs reduce inflammation via inhibiting autophagy. Microglia-derived IL-6 promoted PC12 neuronal apoptosis, as well as induced autophagy via promoting STAT3 phosphorylation and suppressing *miR-30d* that directly targets ATG5 [84]. In addition, *miR-144* directly interacted with mTOR 3′-UTR to inhibit mTOR expression, and further promoted autophagy mediated neuroinflammation in hemoglobin stimulated microglia and perihematomal brain from ICH mice [85,86]. Another study reported that m*iR-23b* overexpression down-regulated pro-inflammatory mediators’ expression in hemin-induced BV2 cells and prevented co-cultured HT22 cells apoptosis via directly targeting inositol polyphosphate multikinase (IPMK) and activating AKT/mTOR pathway to suppress autophagic flux. Further, *miR-23b* overexpression also relieved ICH-triggered brain injury, inhibited neurons apoptosis and microglia activation, and reduced pro-inflammatory factors expression in the brain of ICH rats [87]. Moreover, overexpression of *miR-27a* ameliorated TBI-triggered neurological deficiency, inhibited autophagy via targeting the 3’ UTR of FOXO3a mRNA to suppress the expression of FOXO3a in the hippocampus after TBI rats [88]. To sum up, miRNAs could reduce neuronal apoptosis, promote the degradation of abnormal proteins, and inhibit the production of pro-inflammatory mediators to alleviate CNS diseases by regulating autophagy.

#### 3.1.2 circRNAs and lncRNAs

In methamphetamine-induced neuroinflammation in astrocytes, the expression of GFAP, LC3-II and DDIT3 were increased, which were further aggravated by autophagy inhibitor 3-MA and were reversed by endoplasmic reticulum stress inhibitor salubrinal and autophagy inducer rapamycin. Methamphetamine also inhibited *miR124-2HG* expression and promoted Sigmar1 expression. Nevertheless, *miR124-2HG* down-regulated Sigmar1 gene and protein expression, suppressed autophagy and endoplasmic reticulum stress, and inhibited astrocytes activation in methamphetamine-induced astrocytes. And inhibition of *circHIPK2* that binds *miR124-2HG* had similar effects like *miR124-2HG*. *In vivo*, anti-*miR124-2HG* accelerated the expression of Sigmar1 and GFAP in the hippocampus, which were inhibited by Sigmar1 knockout. Besides, in methamphetamine-induced neuroinflammation mice, *circHIPK2* knockdown also suppressed astrocytes activation and restrained Sigmar1 and GFAP proteins expression [89]. Additionally, *circRS-7* inhibition reduced chronic constriction injury (CCI)-triggered neuropathic pain, inhibited microglia activation, reduced the production of pro-inflammatory cytokines, and suppressed autophagy via targeting *miR-135a-5p* in CCI rats [90]. The above studies indicate that circRNAs inhibit the production of related miRNAs to regulate autophagy and thus reduce inflammation in CNS diseases.

Long intergenic noncoding RNA Cox2 (*lincRNA-Cox2*) knockdown decreased IL-1β level, prevented NLRP3 inflammasome activation, and increased LC3-II/I ratio that were further up-regulated by NLRP3, ASC and Caspase-1 siRNA, as well as weakened by ATG5 and TIR-domain-containing adapter-inducing interferon-β (TRIF) siRNA via suppressing NF-κB signal in LPS-induced microglia cells. And *in vivo*, *lincRNA-Cox2* knockdown attenuated EAE injure and neuroinflammation, while inducing autophagy of microglia in the spinal cords from EAE mice [91]. Furthermore, in MPP^+^-induced SK-N-SH cells, *lncRNA-HOTAIR* knockdown reversed MPP^+^-induced cell damage, including enhanced cell viability, inhibition of apoptosis, as well as the down-regulation of pro-inflammatory mediator levels. Absence of *lncRNA-HOTAIR* also up-regulated *miR-874-5p* expression and reduced ATG10 protein expression, while the effects of HOTAIR knockdown on MPP^+^-induced neuronal injury were reversed by *miR-874-5p* inhibition [92]. In summary, lncRNAs might reduce inflammation in neurological diseases by regulating autophagy to inhibit the production of pro-inflammatory mediators, activation of NLRP3 inflammatory vesicles and apoptosis.

### 3.2 Histone Modification

Histone deacetylases inhibitor, suberoylanilide hydroxamic acid increased the levels of H3 and H4 histone acetylation and the ratio of LC3-II/I, and reduced p62 expression in sevoflurane-exposed primary neurons. Histone deacetylases inhibitor also improved cognitive impairment, up-regulated histone acetylation levels, ameliorated autophagy impairments and suppressed the activation of NLRP3 inflammasome in the hippocampus from sevoflurane-stimulated aged mice [93]. Moreover, anacardic acid, a histone acetyltransferases inhibitor, decreased pro-inflammatory mediators’ levels, suppressed autophagy and down-regulated the expression of H3K9ac in calcitonin gene-related peptide (CGRP)-stimulated astrocyte cells. And in CCI rats, CGRP antagonist and histone acetyltransferases inhibitor prevented neuropathic pain and astrocyte activation and inhibited the expression levels of H3K9ac in the spinal dorsal horn [94]. In summary, histone modification may inhibit the activation of glial cells and NLRP3 inflammasome through regulating autophagy, as amply shown in Table 1.

## 4. Conclusion and perspectives

**Figure 2.**
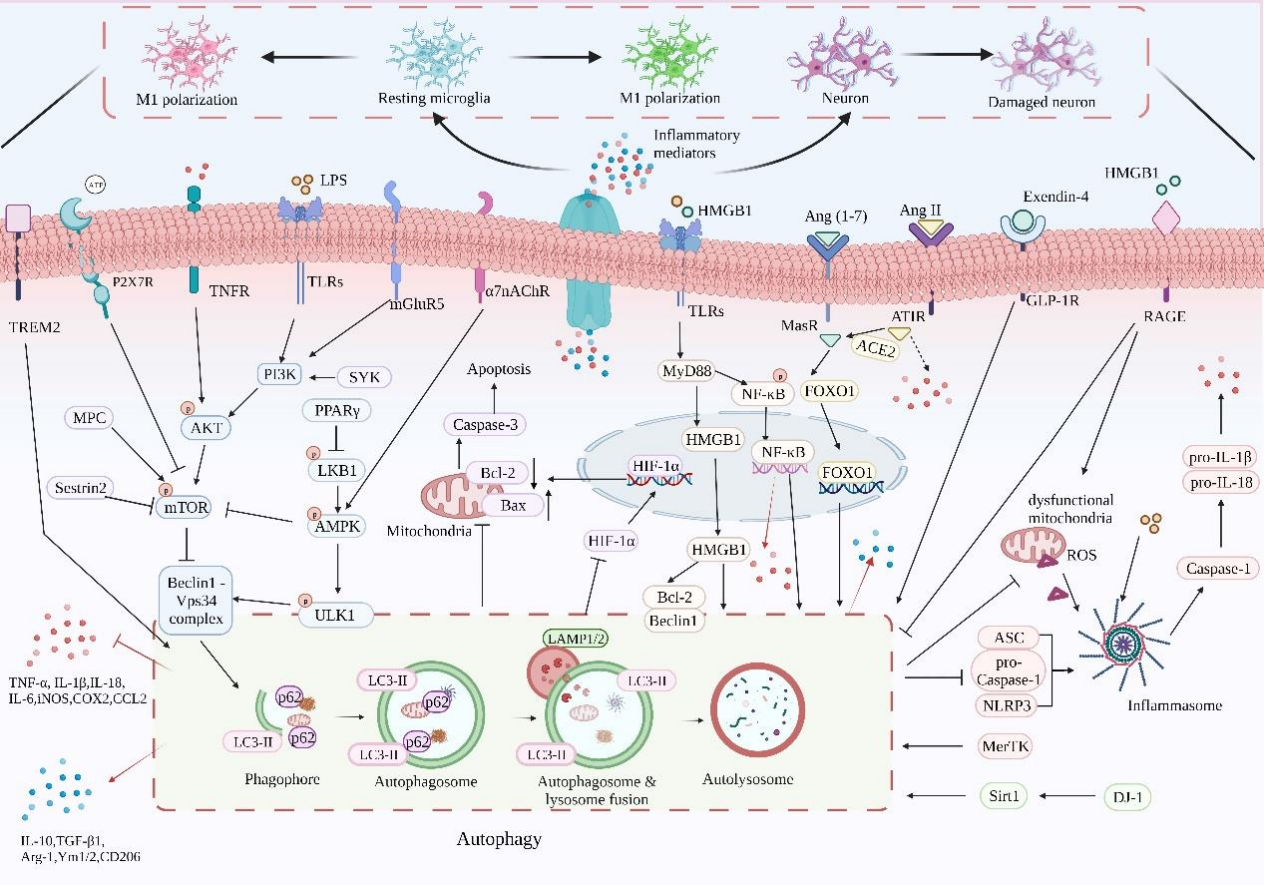
**Overview of the mechanisms of autophagy in neuroinflammation**. The inhibitory effect of autophagy in neuroinflammation involves multiple signaling pathways, including cellular metabolism, apoptosis and inflammasome.

**Figure 3.**
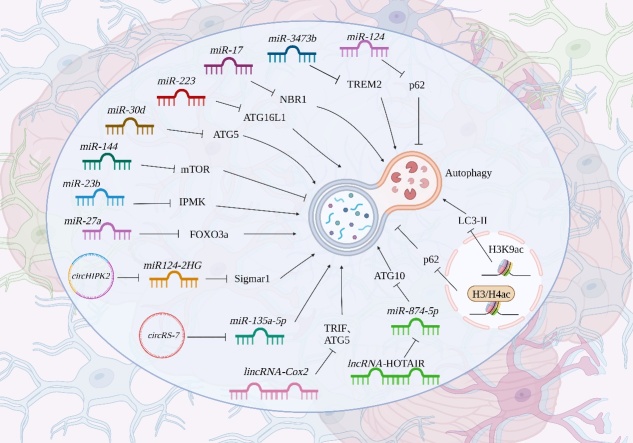
**Epigenetic regulation of autophagy in neuroinflammation**. Autophagy in neuroinflammation is influenced by epigenetic modifications, including non-coding RNAs and histone modification.

Inflammation in the CNS is a hallmark of neurological diseases, and therapeutic strategies targeting neuroinflammation have been extensively investigated. Autophagy, a tightly controlled cellular decomposition pathway, eliminates pathogens, damaged organelles and other cargos to maintain the homeostasis of the intracellular environment under various stimuli. Autophagy could influence CNS diseases by modulating neuroinflammation. According to recent studies, numerous signals, including cellular metabolism, apoptosis and inflammasome, are involved in the effects of autophagy on neuroinflammation (Fig. 2). Specifically, enhancement of autophagy through inhibiting mTOR pathway and promoting AMPK and FOXO1 pathways is able to suppress microglia activation, facilitate M2 microglia polarization, promote the production of anti-inflammatory mediators, reduce the levels of pro-inflammatory mediators, alleviate apoptosis and inactivate NLRP3 inflammasome, thereby treating a variety of CNS diseases, such as AD, PD and TBI. Additionally, since epigenetic deficiencies happen during the early stages of CNS disorders, interference methodologies targeting epigenetic modifications have been proposed as prevention strategies. Notably, epigenetic modifications also affect autophagy in neuroinflammation, particularly non-coding RNAs and histone acetylation, which may adjust autophagy-related genes, thus impacting their transcription and subsequent autophagy to alleviate inflammation in CNS diseases (Fig. 3).

Despite extensive research on the regulation of autophagy in neuroinflammation and related CNS diseases, there are still some limitations of the current studies. Firstly, the role of autophagy on neuroinflammation in AD, PD, TBI and cerebral ischemia attract more investigators, and other CNS diseases such as multiple sclerosis, migraine and ICH have received less attention, which need to be further studied. Secondly, current reports mainly focus on microglia, with less researches on other important brain cells such as astrocytes and neurons. Thirdly, the mechanisms by which autophagy regulates neuroinflammation are mainly concerned with mTOR and NLRP3 inflammasome, while other pathways are less well studied. In addition, epigenetic modifications, mainly including DNA methylation, histone modifications, and non-coding RNAs, modulate neuroinflammation and related brain diseases [95, 96]. However, currently, non-coding RNAs and histone modification have received extensive attention and research from scholars on autophagy regulation of neuroinflammation and DNA methylation have hardly been reported. DNA methylation mediates transcriptional silencing of downstream genes through recruitment of repressive transcription factors, which in turn leads to reduce gene and protein expression [97]. DNA methylation may also regulate neuroinflammation and associated CNS disorders by regulating the expression of autophagy-related genes and proteins, which need to be further studied. Overall, these data indicate that the development of therapeutic agents that specifically target the epigenetic mechanisms of autophagy and the signaling connected to autophagy will have a significant impact on the treatment of CNS diseases linked to neuroinflammation.
